# Biological potential alterations of migratory chondrogenic progenitor cells during knee osteoarthritic progression

**DOI:** 10.1186/s13075-020-2144-z

**Published:** 2020-03-27

**Authors:** Yu-Xing Wang, Zhi-Dong Zhao, Qian Wang, Zhong-Li Li, Ya Huang, Sen Zhao, Wei Hu, Jia-Wu Liang, Pei-Lin Li, Hua Wang, Ning Mao, Chu-Tse Wu, Heng Zhu

**Affiliations:** 1 People’s Liberation Army General Hospital, Road Fuxing 28, Beijing, 100853 People’s Republic of China; 2 Beijing Institute of Radiation Medicine, Road Taiping 27, Beijing, 100850 People’s Republic of China; 3 Beijing Institute of Basic Medical Sciences, Road Taiping 27, Beijing, 100850 People’s Republic of China

**Keywords:** Knee osteoarthritis, Degenerative cartilage, Migratory chondrogenic progenitor cells, Chondrogenic differentiation

## Abstract

**Background:**

Although increasing studies have demonstrated that chondrogenic progenitor cells (CPCs) remain present in human osteoarthritic cartilage, the biological alterations of the CPCs from the less diseased lateral tibial condyle and the more diseased medial condyle of same patient remain to be investigated.

**Methods:**

CPCs were isolated from paired grade 1–2 and grade 3–4 osteoarthritic cartilage by virtue of cell migratory capacities. The cell morphology, immunophenotype, self-renewal, multi-differentiation, and cell migration of these CPCs were evaluated. Additionally, the distributions of CD105^+^/CD271^+^ cells in OA osteochondral specimen were determined. Furthermore, a high-throughput mRNA sequencing was performed.

**Results:**

Migratory CPCs (mCPCs) robustly outgrew from mildly collagenases-digested osteoarthritic cartilages. The mCPCs from grade 3–4 cartilages (mCPCs, grades 3–4) harbored morphological characteristics, cell proliferation, and colony formation capacity that were similar to those of the mCPCs from the grade 1–2 OA cartilages (mCPCs, grades 1–2). However, the mCPCs (grades 3–4) highly expressed CD271. In addition, the mCPCs (grades 3–4) showed enhanced osteo-adipogenic activities and decreased chondrogenic capacity. Furthermore, the mCPCs (grades 3–4) exhibited stronger cell migration in response to osteoarthritis synovial fluids. More CD105^+^/CD271^+^ cells resided in grade 3–4 articular cartilages. Moreover, the results of mRNA sequencing showed that mCPCs (grades 3–4) expressed higher migratory molecules.

**Conclusions:**

Our data suggest that more mCPCs (grades 3–4) migrate to injured articular cartilages but with enhanced osteo-adipogenic and decreased chondrogenic capacity, which might explain the pathological changes of mCPCs during the progression of OA from early to late stages. Thus, these dysfunctional mCPCs might be optional cell targets for OA therapies.

## Introduction

Knee osteoarthritis (KOA) is one of the most common degenerative disorders in joints and has been anticipated to be the fourth leading cause of disability worldwide by the year 2020 [1, 2]. It is mainly characterized by slowly progressive degeneration and loss of the articular cartilage. Unfortunately, incomplete understanding of the pathogenesis of KOA confined the development of therapeutic strategies, and there are few curable treatments available so far for osteoarthritis (OA) until the end stage of the disease necessitates joint replacement [3, 4].

In the past decades, articular cartilages have been considered as hypocellular and hypovascular tissues and possessed poor capacities to self-repair. Promisingly, recent investigations have shown the normal and OA articular cartilages containing tissue-specific stem/progenitor cells, named chondrogenic progenitor cells (CPCs), with high proliferative, clonogenic, and multi-differentiation capacities [5, 6]. In addition, CPCs are capable of migrating to injured sites after cartilage trauma [7] or diseased osteoarthritic cartilages [8]. Furthermore, CPCs have recently attracted interest due to their immunoregulatory properties [9, 10] and phagocytic capacity [11], which have been suggested as valuable potentials for cell-based therapies [6, 12–14]. However, numerous studies from independent teams brought inconclusive information in understanding CPCs activity at different phase of knee OA progression. Seol et al. reported that CPCs represented a transient emergence and homing after cartilage mechanical injuries [7]. In addition, Tong et al. showed that CPCs harbored a transient proliferative response in early OA and became gradually quiet as OA progresses [15]. Nevertheless, the pathological changes of CPCs during the development of OA and the biological mechanisms governing these cells remain to be elucidated.

Fortunately, a portion of OA patients with total knee arthroplasty (TKA) present Outerbridge grade 3–4 cartilage lesions in the medial compartment accompanied by grade 1–2 cartilage lesions in the lateral side [16, 17], which provide an opportunity to explore the CPC changes in different grades of osteoarthritic cartilage in a strictly matched manner so as to avoid individual heterogeneity [18]. Xia al. investigated the relative cell percentage, proliferation activity, multi-lineage differentiation potential, and miRNA expression profile of a subpopulation of human CPCs with CD105 and CD166 co-expression by isolating cells from the degraded cartilages that are in the medial condyle and relatively normal cartilage on the lateral side [19]. In addition, CPCs derived from paired grade 1–2 cartilage on the lateral femoral condyle and grade 3–4 cartilage on the medial femoral condyle were assayed by a standardized colony-forming unit assay by using automated image analysis software [18]. However, all of the CPCs were obtained from the released cells post collagenase digestion either by cell colony formation cell expansion or flow cytometry cell sorting [20, 21]. In addition, most of these cells were obtained from femoral condyles instead of from the osteoarthritic tibial plateau cartilages which usually underwent significant pathological changes during OA progression.

Our previous study reported an effective strategy of isolating functional CPCs from human articular cartilages by virtue of cell outgrowth after a short-time collagenase digestion [12], which are supposed to accelerate cell migration with only little proteoglycan loss in the edge of tissue and minimal cell death [22]. This subpopulation of CPCs exhibited high cell proliferation and cartilage regenerative capacity than that of released cells, which may benefit from mimicking the stem/progenitor niche in vitro [12, 23]. Therefore, we hypothesized that culturing the short-time collagenase-digested OA cartilage fragments may obtain novel subpopulations of CPCs, which may be helpful to understand the CPC changes during progression of KOA. In the current study, we cultured CPCs from paired grade 1–2 OA on the lateral tibial plateau and grade 3–4 OA on the medial tibial plateau cartilage from the same donor by virtue of cell migrations. The CPC immunophenotype, self-renewal, multi-differentiation, cell migration, in vivo distribution, and gene expression in the CPCs were also investigated.

## Methods

### Patient characteristics

This study was approved by the institutional ethical review board of our Hospital (Rapid review of scientific research projects for use of discarded biological material), and informed consent was obtained from all donors. Twenty-eight patients (9 male and 19 female, mean age, 63.6 years [range, 53–73 years]; mean body mass index, 26.0 kg/m^2^ [range, 22.7–30.8 kg/m^2^]; mean disease duration 7.3 years [range, 3–15 years]) (Supplementary Table 1) who were diagnosed with late-stage idiopathic KOA according to the criteria of the American College of Rheumatology [24] with varus malalignment of the lower extremity and scheduled for elective TKA were recruited. Radiographs exhibited a relatively spared lateral femoral compartment (joint space > 3 mm). Cartilage morphology was scored according to the whole-organ magnetic resonance imaging score [25] (mean cartilage scores 19.4 [range, 12.0–25.0] for medial femorotibial joint; mean cartilage scores 6.2 [range, 3.0–10.0] for lateral femorotibial joint) (Supplementary Table 1). Patients were excluded if they had secondary arthritis related to systemic inflammatory arthritis or if their history included previous systemic or intraarticular injection glucocorticoids, prior ipsilateral knee surgery, knee injury, infection, or osteonecrosis. There are 28 patients including in our study. Eighteen patient specimens were used for histopathology experiments, and 10 other patient specimens were used for cell isolation. Among 18 patient specimens, 6 patient specimens were too hard to completely decalcify; thus, 12 were used for HE staining, and among them, 8 randomly selected patient specimens were used for CD105/CD271 staining.

### Isolation, expansion, and identification of mCPCs

**Fig. 1:**
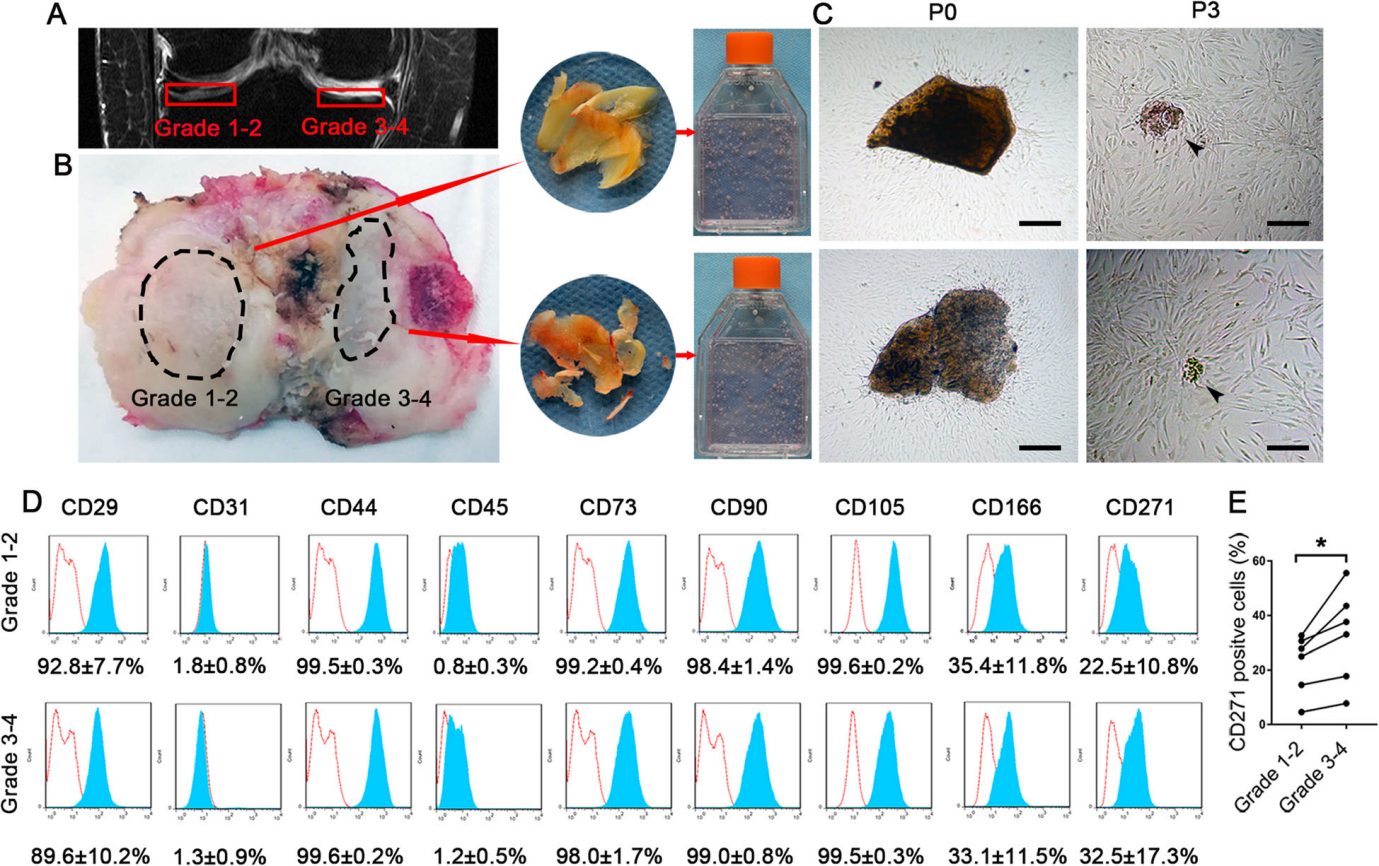
Isolation, expansion, and immunophenotyping of mCPCs from paired grade 1–2 and grade 3–4 OA cartilage. **a** Representative MRI image of late-stage idiopathic KOA shows Outerbridge grade 3–4 cartilage lesions in the medial tibial plateau and grade 1–2 cartilage lesions on the lateral side. **b** Osteoarthritic cartilage specimen of the tibial plateau obtained from the same patient. The cartilages from marked areas were harvested. The cartilaginous tissues were separated from the subchondral bone, minced into pieces, mildly digested, and incubated in culture medium. **c** mCPCs migrated from paired OA cartilage pieces after 15 days of cultivation. The cartilage pieces were retained and maintained until passage 3 (arrowhead). **d** Immunophenotype markers (CD29, CD31, CD44, CD45, CD73, CD90, CD105, CD166, and CD271) of mCPCs from paired grade 1–2 and grade 3–4 cartilage. Red lines indicate isotype controls. **e** The expression of CD271 in mCPCs from grade 1–2 cartilage was significantly lower compared with those from grade 3–4 cartilage (*n* = 6 donors, with each repeated in replicates, *p* = 0.034). Scale bars in **d** represent 200 μm. mCPCs, migratory chondrogenic progenitor cells; KOA, knee osteoarthritis

During the arthroplasty procedure, an osteochondral specimen of the tibial plateau was harvested with the initial proximal tibial cut. Samples of Outerbridge grade 1–2 cartilage were obtained from the lateral tibial plateau, and samples of grade 3–4 cartilage were obtained from the medial tibial plateau (*n* = 10 donors). Grade 1–2 cartilage includes cartilages with an intact surface (grade 1) and minimal fibrillation (grade 2), and grade 3–4 cartilage includes cartilage with fissures to subchondral bone [26]. The methods used to harvest the CPCs have been described in previous studies [5, 12, 27, 28] with minor modifications. In brief, the cartilaginous tissues were separated from the osteoarthritic articular cartilages without contaminated subchondral bones and were minced into pieces (about 1.0 mm × 1.0 mm × 1.0 mm, Fig. 1b), and then digested in 0.1% collagenase II (Sigma) for 2 h. The released cells were abandoned, and the digested cartilage chips were incubated in alpha-minimal essential medium (α-MEM) with 10% vol/vol fetal bovine serum (FBS) (Invitrogen Life Technologies) at 37 °C in an atmosphere of 5% CO_2_. The mCPCs outgrew from cartilage chips within 10 days, and the adhesive cells rapidly reached 60–80% confluence in another 5 days. Importantly, the cartilage chips were retained and maintained until passage 3 to mimic the stem/progenitor niche ex vivo and allow more CPC outgrowth. The morphological characteristics of CPCs were observed with reverted light microscope (Olympus BX71).

### Flow cytometry analysis

The cell surface antigen profile of paired mCPCs (*n* = 6 donors) was analyzed by flow cytometry. mCPCs at passage 3 were harvested by trypsin digestion, and antibodies were stained individually (phycoerythrin (PE)-conjugated monoclonal antibodies against human CD29, CD44, CD73, and CD166; fluorescein isothiocyanate (FITC)-conjugated monoclonal antibodies against human CD45, CD90, and CD271; and allophycocyanin (APC)-conjugated antibodies against CD31 and CD105; eBioscience) for 30 min in the dark at 4 °C. After 2 washes with phosphate-buffered saline (PBS), events were collected by flow cytometry with a FACScalibur system (BectoneDickinson), and the data were analyzed using FlowJo 7.6 software.

### Growth kinetics and CCK-8 assay

The growth kinetics was determined using the trypan blue exclusion cell count method for hemocytometer cell counting [29]. Briefly, paired mCPCs (*n* = 6 donors) were cultured in 48-well plates at 2 × 10^4^ cells/well and harvested every 3 days for hemocytometer cell counting during a period of 19 days. The Cell Counting Kit 8 (CCK-8, Dojindo, Japan) assay was conducted according to a previous study [30]. In brief, CPCs at passage 4 were seeded in 96-well plates (1 × 10^3^ cells/well, 5 wells in each group) and maintained in culture medium, and the CCK-8 solution was added at a ratio of 100 μl/ml and incubated at 37 °C for 1 h. The absorbance was measured at a wavelength of 450 nm on days 1, 4, 7, 10, 13, 16, and 19.

### Colony-forming unit fibroblast formation (CFU-F) assay

Passage 4 paired mCPCs (*n* = 6 donors) in each group were adjusted to different cell numbers (1 × 10^3^ and 5 × 10^3^ cells/well). Aliquots of cell suspensions were added to 6-well culture plates and were maintained in culture for 10 days. Crystal violet was used to stain the colonies, and their vertical gross appearances were imaged by digital photography.

### Evaluation of the multi-potency of mCPCs

Osteogenic, adipogenic, and chondrogenic differentiation was assayed. The previously reported protocols for CPC differentiation were used with minor revision in the current study [23]. Briefly, for osteogenic differentiation, cells were harvested and incubated in osteogenic induction medium (10 mM of glycerol-2-phosphate, 0.1 μM of dexamethasone, and 20 μM of ascorbic acid) for 14 or 28 days. The osteogenic activity was assessed at day 14 by alkaline phosphatase (ALP) staining and at day 28 by Von Kossa staining, respectively. For adipogenic differentiation, CPCs were cultivated at 1 × 10^4^ cells/well in 48-well cell culture plates, adipogenic induction medium (1 μM of isobutylmethylxanthine and 10^− 3^ μM of dexamethasone) was supplemented, and Oil Red O staining (day 14) was performed to assess the adipogenic potency. For chondrogenic differentiation, 4 × 10^5^ CPCs were centrifuged in polypropylene tubes to form a pelleted micromass and maintained in chondrogenic induction medium consisting α-MEM supplemented with 10^7^M of dexamethasone, 1% (vol/vol) insulin-transferrin-sodium selenite, 50 μM of ascorbate-2 phosphate, 1 mM of sodium pyruvate, 50 μg/ml (wt/vol) of proline, and 20 ng/ml (wt/vol) of transforming growth factor (TGF-β_3_). On day 28, the pellets were fixed and sectioned. The development of chondrocytes and accumulation of the cartilage matrix were evaluated by hematoxylin eosin, toluidine blue, and Safranin O staining. The expressions of Sox-9 (SRY-type high-mobility group box-9) and Col-II (collagen type II) were detected by immunohistochemical assays. The images were captured using a microscope under brightfield mode. Chondrogenesis was also analyzed according to a previously published histological pellet scoring system [31]. Data were obtained from six paired samples, with each repeated in triplicates.

### Histologic and immunohistochemical analysis

The osteochondral specimens of initial proximal tibial cut during the arthroplasty procedure were also collected for histologic immunohistochemical analysis (*n* = 18 donors). Samples were placed in 10% formalin before processing. For each patient, separate lateral and medial tibial plateau pieces were decalcified using 10% ethylenediaminetetraacetic acid (EDTA, Sigma) for 3–4 months and then mounted on paraffin blocks. Decalcified tissue specimens were stained with hematoxylin and eosin. Immunohistochemistry for CD105 and CD271 (NGF receptor) staining was performed. Mouse anti-human CD105 and CD271 monoclonal antibody (Abcam) was used at a dilution of 1:50. Digital image analysis was performed to evaluate relative cartilage damage (including the cartilage–bone interface) and CD105^+^ and CD271^+^ cells in vivo distribution. For each sample, the whole tissue area was scanned using an OlympusX71 microscope under brightfield mode depending on the size of the section; 2–5 images were captured for the cartilage area (including the cartilage–bone interface).

### OA synovial fluid-mediated migration of mCPCs

Migration of paired mCPCs (*n* = 5 donors) on stimulation with OA pro-inflammatory synovial fluid [32] (from three symptomatic idiopathic KOA patient) was analyzed in 24-well transwell plates (8 μm pore size of polycarbonate membranes, Corning) as described previously [33]. In brief, 5 × 10^4^ mCPCs in serum-free α-MEM medium were seeded in the upper wells. The lower wells were filled with 0%, 20%, and 40% OA synovial fluid. After being incubated at 37 °C for 10 or 20 h, cells that migrated through the polycarbonate membrane were fixed with acetone/methanol (1:1, vol/vol). Non-migrating cells on top of the membrane were removed. Migrated cells were stained by 4′,6-diamidino-2-phenylindole (DAPI) and crystal violet and counted microscopically. Three representative photographs (left, right, and central) of each well were taken, migrated cells per picture were counted using Image J (National Institutes of Health, Bethesda, MD), the total number of migrated cells was extrapolated to the total well, and the migration rates were calculated.

### Real-time quantitative polymerase chain reaction (RT-qPCR)

RT-qPCR was performed to further evaluate their multi-lineage differentiation and RNA sequencing validation. After maintaining in osteogenic, adipogenic, and chondrogenic differentiation media at a density of 5 × 10^4^ cells/well in 6-well cell culture plates for 10 days, the total RNA was extracted using Trizol reagent (Fermentas) and reverse transcribed using an mRNA Selective PCR Kit (TaKaRa) according to the manufacturer’s instructions. Human runt-related transcription factor 2 (RUNX2), osteocalcin (OCN), CCAAT/enhancer-binding protein alpha (CEBP/α), peroxisome proliferator-activated receptor gamma (PPARγ), sex-determining region Y-box 9 (Sox-9), and collagen type II (Col-II) cDNA were amplified by real-time PCR using a SYBR PCR Master Mix Kit (Sigma-Aldrich). The primers were synthesized by Invitrogen (Shanghai, China), and the sequences are shown in Supplementary Table 2. The mRNA levels were normalized to the value of β-actin or RPL13a (housekeeping genes for Sox-9 and Col-II only) [34]. Mean fold changes were calculated. Data presented are the mean of the six different donors, with each repeated in triplicates.

### mRNA expression profile of mCPCs by RNA sequencing analysis

We used equal amounts of total RNA from each of 6 patients’ paired mCPCs from grade 1–2 and grade 3–4 osteoarthritic cartilage. The RNA sequencing was performed by GeneWIZ Technology (Suzhou, China). Briefly, the quality control of gene expression profile analysis was performed by using Agilent bioanalyzer 2100 system. The mRNAs were captured by NEBNext Poly(A) mRNA magnetic isolation module. Library construction was conducted by NEBNext ultra RNA library RNA PREP kit for Illumina. Library purification was conducted by Beckman Agencourt AMPure XP beads. Library quantification and results verification were performed by Agilent bioanalyzer 2100 and Qubit system. CBOT clustering and Hiseq were respectively performed by using TruSeq PE cluster Kit V4 and TruSeq SBS Kit V4-HS. Bioinformatics analysis was performed according to the manufacturer’s protocols. We then selected relative expression of genes associated with OA pathogenesis (involved in mesenchymal stem cell [MSC] tripotentiality, collagen metabolism, chemotaxis, angiogenesis, and control of osteoclast activation and other genes). We clustered the significantly increased and decreased genes according to various biological processes, cellular component, and molecular function and analyzed the differentially expressed genes in the Kyoto Encyclopedia of Genes and Genomes (KEGG) pathways [35]. Expressions of arbitrarily selected dysregulated genes were validated by RT-qPCR (*n* = 6 donors).

### Statistical analysis

Data were presented as mean values and standard deviation (SD). The normal distribution of data was confirmed with the Kolmogorov-Smirnov test. As for normally distributed data (flow cytometric measurements, CFU-F assays, growth kinetic parameter, gene expression, and migration rates between mCPCs from paired grade 1–2 and grade 3–4 cartilage), a paired *t* test was employed; for ordinal grading data such as the pellet histological scores, a Wilcoxon signed-rank test was applied. A *p* value < 0.05 was considered statistically significant. All tests were performed using IBM SPSS Statistics 20.0.

## Results

### The morphological characteristics of mCPCs of knee OA patients

The cartilaginous tissues for CPC culturing were harvested from the articular cartilages of knee OA patients (Fig. 1a, b). Approximately 10 days after the primary culture, fibroblast-like cells migrated out from the digested cartilage fragments and adhered to the plastic dishes in both grade 1–2 and grade 3–4 groups (Fig. 1c). More out-migrated cells can be seen around the grade 3–4 OA cartilage fragments than the grade 3–4 OA cartilage fragments in the same isolation and culture system (Fig. 1c). A primary culture confluence of 60–80% was usually achieved within 15 days. The cell morphology of mCPCs is macroscopically identical. An adherent layer of vortex-shaped cells developed, and cartilage pieces can still be seen within 28 days at P3 (Fig. 1c).

### mCPCs from grade 3–4 cartilage highly expressed CD271

The results of immunophenotyping showed that mCPCs from both grade 1–2 and grade 3–4 cartilage were homogenously negative for CD34, and CD45, and positive for CD29, CD44, CD73, CD90, CD105, and CD166 expressions (Fig. 1d). However, the expression of CD271 was significantly higher in grade 3–4 cartilage (32.5 ± 17.3%) in comparison with grade 1–2 cartilage (22.5 ± 10.8%) (*p* = 0.034) (Fig. 1e).

### The mCPCs (grade 3–4) exhibited similar proliferation potential and self-renewal capacity to that of mCPCs (grade 1–2)

To investigate the proliferation ability, hemocytometer cell counting and a CCK-8 assay were performed. The results of the hemocytometer cell counting proliferation assay (Supplementary Fig. 1A and B) showed that mCPCs in both grade 1–2 and grade 3–4 cartilage exerted similar proliferation ability (*p* > 0.05). Consistently, similar cell proliferation is also reflected by the CCK-8 assay (Supplementary Fig. 1C). Self-renewal potential was measured in a CFU-F assay. mCPCs in both grade 1–2 and grade 3–4 cartilage performed similarly with comparable clonogenic ability (Supplementary Fig. 1D and E).

### mCPCs from the grade 3–4 OA cartilage showed stronger osteogenic, adipogenic, and weaker chondrogenic potential

**Fig. 2:**
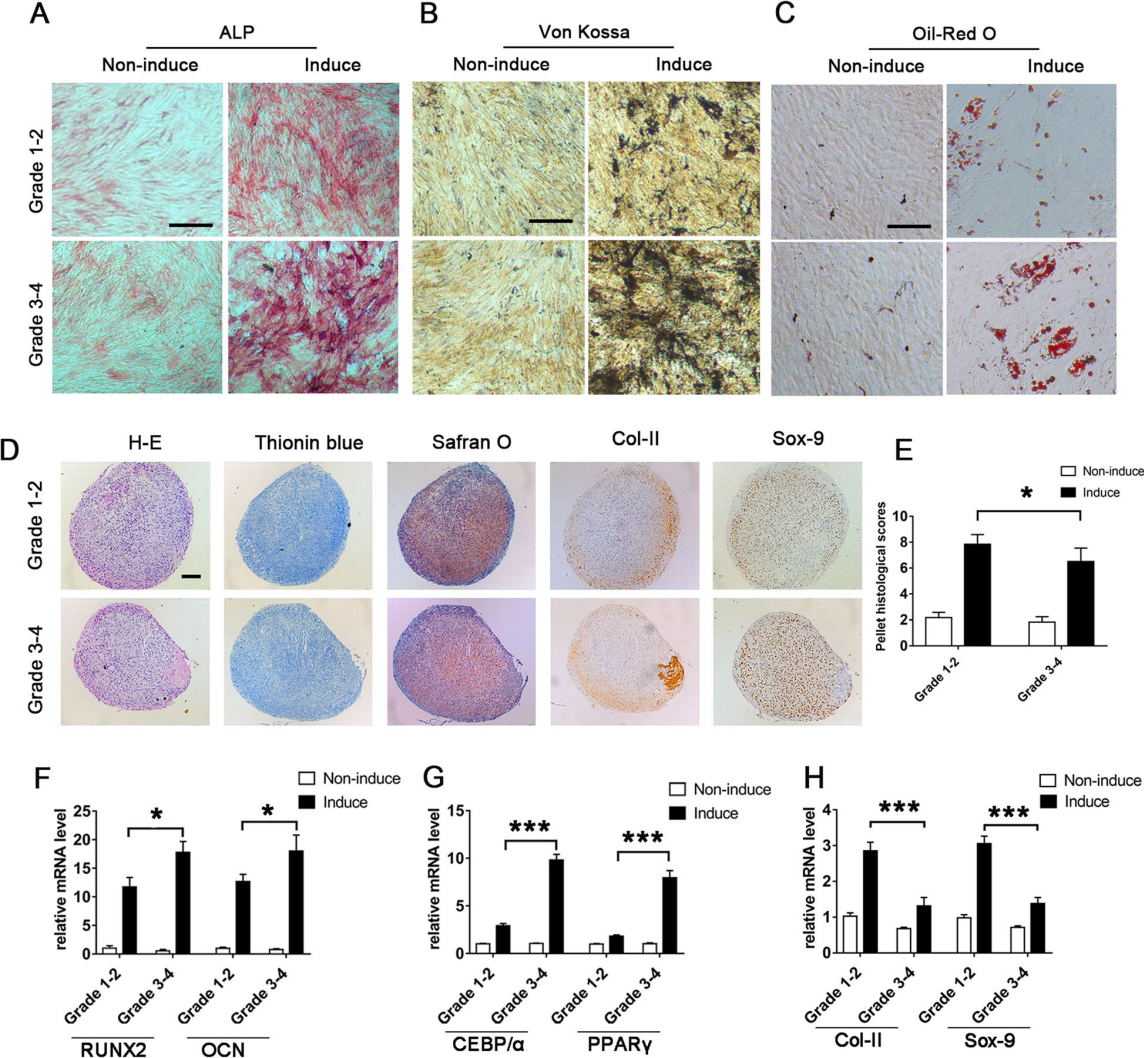
Multi-differentiation of mCPCs derived from paired grade 1–2 and grade 3–4 OA cartilage. **a**–**c** Representative photomicrographs of ALP (**a**), Von Kossa (**b**), and Oil Red O (**c**) staining of mCPCs. **d** Histochemical staining of HE, toluidine blue, Safranin O, and immunohistochemical staining of Col-II and Sox-9 for cartilage pellets that developed with paired grade 1–2 and grade 3–4 cartilage-derived mCPCs. **e** Histological scores of pellets. **f**–**h** The relative mRNA expression of osteogenesis (RUNX2 and OCN), adipogenesis (CEBP/α and PPARγ), and chondrogenesis genes (Col-II and Sox-9). Data were normalized to β-actin and RPL13a (housekeeping genes for Sox-9 and Col-II only). *n* = 6 donors, with each repeated in triplicates, error bars denote the means ± SD, *p < 0.05, ***p < 0.001. Scale bars represent 200 μm (**a**–**c**) and 100 μm (**d**), respectively. ALP, alkaline phosphatase; HE, hematoxylin and eosin; RUNX2, runt-related transcription factor 2; OCN, osteocalcin; CEBP/α, CCAAT/enhancer-binding protein alpha; PPARγ, peroxisome proliferator-activated receptor gamma; Col-II, collagen type II; Sox-9: sex-determining region Y-box 9

mCPCs from both grade 1–2 and grade 3–4 cartilage were able to differentiate toward the osteogenic, adipogenic, and chondrogenic lineage. In particular, mCPCs in grade 3–4 cartilage display enhanced osteo- and adipogenic differentiation capacity compared to mCPCs in grade 1–2 cartilage. mCPCs from grade 3–4 cartilage exhibited higher ALP activity (Fig. 2a) and higher amount of calcium deposition (Fig. 2b) than that of CPCs from grade 1–2 cartilage. Also, mCPCs from grade 3–4 cartilage harbored higher amount of intracellular Oil Red O-stained lipids than that of mCPCs from grade 1–2 cartilage (Fig. 2c). Consistent with the results of cytochemical staining analysis, mCPCs from grade 3–4 cartilage after differentiating induction exhibited high levels of mRNA expression of osteogenic markers (RUNX-2 and OCN) (Fig. 2f) and adipogenic transcription factors (CEBP/α and PPARγ) (Fig. 2g) than that of mCPCs from grade 1–2 cartilage. However, the results of HE, toluidine blue, and Safranin O staining as well as immunostaining of Col-II and Sox-9 showed that mCPCs from grade 3–4 cartilage exhibited less capacity of pellets formation than their counterparts from grade 1–2 cartilage (Fig. 2d, e). In addition, the expression of chondrogenic transcription factors Col-II and Sox-9 further confirmed the decreased chondrogenic capacity of mCPCs from grade 3–4 cartilage (Fig. 2h).

### mCPCs from the grade 3–4 OA cartilage showed stronger migration potential

**Fig. 3:**
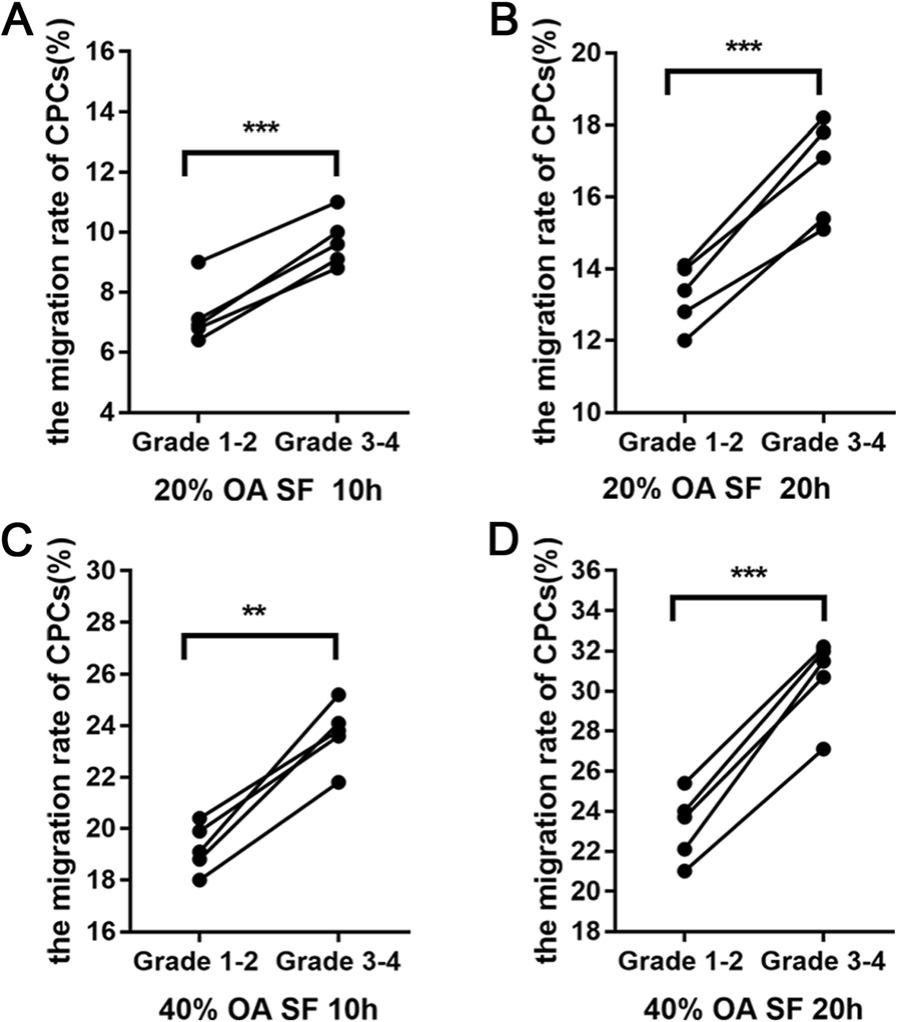
Migration potential of mCPCs from paired grade 1–2 and grade 3–4 OA cartilage. **a**–**d** In the presence of graded concentration of OASF (20% and 40% OA), grade 3–4 OA cartilage-derived mCPCs exhibited higher migration rates than that of their counterpart from grade 1–2 OA cartilage at 10 h and 20 h (*n* = 5 donors, with each repeated in triplicates). **p < 0.01, ***p < 0.001. OASF, osteoarthritis synovial fluid

mCPCs from both grade 1–2 and grade 3–4 cartilage were cultivated in the presence of OA synovial fluid (OASF, 20% and 40%) for 10 h or 20 h. Cell culture medium without OASF serves as negative control. The results of crystal violet and DAPI staining showed that only a few CPCs passed the bottom of the transwell chambers in the absence of OASF. However, CPCs from both grade 1–2 and grade 3–4 cartilage showed strong migratory activities upon the stimulation of OASF (20% and 40%) (Fig. 3a–d, and Supplementary Fig. 2). In addition, the migration rate of mCPCs from the grade 3–4 OA cartilage was remarkably higher than that of mCPCs from the grade 1–2 OA cartilage in a time-dependent and OASF concentration-dependent manner (Fig. 3a–d, and Supplementary Fig. 2).

### In vivo distribution of CD271^+^ and CD105^+^ cells in grade 1–2 and grade 3–4 OA cartilage

**Fig. 4:**
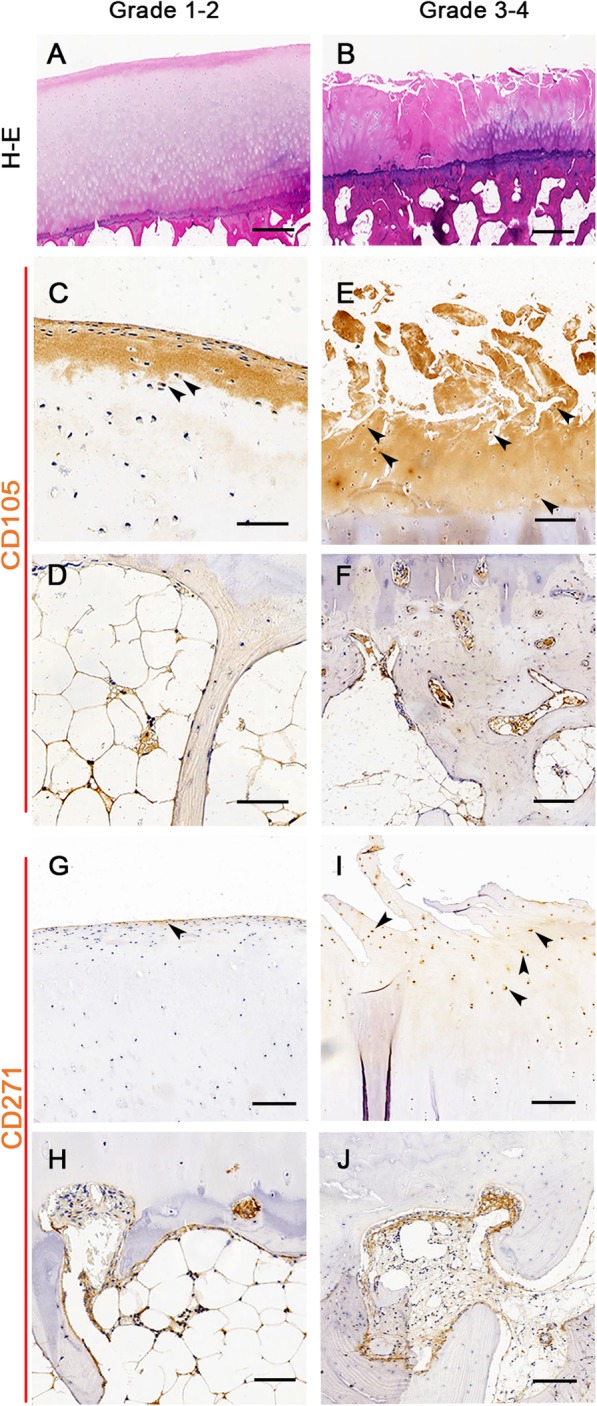
The in situ distribution of CD105^+^ and CD271^+^ cell in paired grade 1–2 and grade 3–4 OA cartilages. **a**, **b** The general morphological characteristics of paired human knee osteoarthritic cartilages were showed by HE staining (*n* = 12 donors). **c**–**f** The results of CD105-targeted immunohistochemical staining showed that more CD105^+^ cells remained present in the grade 3–4 OA cartilage and the bone lining locations near the osteochondral junction area as showed by the arrow (*n* = 8 donors). For CD271^+^ cell distribution, only a few CD271^+^ cells were observed in the superficial cartilage surface of grade 1–2 OA cartilage (**g**). However, more CD271^+^ cells were found in the superficial cartilage surface of grade 3–4 OA cartilage as showed by the arrow (**i**). In addition, CD271^+^ cells distributed near the osteochondral junction regions and reticular pattern of subchondral bone marrow cavities in both grade 1–2 and grade 3–4 OA subchondral bones (**h** and **j**). Scale bars represent 500 μm (**a**, **b**) and 100 μm (**c**–**h**), respectively

We performed histologic assessment of osteochondral specimens from grade 1–2 and grade 3–4 human knee OA articular tissues (*n* = 18 donors). Following decalcification, 12 of 18 paired tissue samples had sufficient tissue quality to enable histologic analysis. Grade 1–2 cartilage showed relatively normal osteochondral structure, with or without slight fibrillation (Fig. 4a), while grade 3–4 cartilage was characterized by fissured or denuded surface with chondrocyte clusters, multiple tidemarks, and thicken trabecular area in the subchondral bone (Fig. 4b). In addition, we investigated the distribution of CD105^+^ and CD271^+^ cells in osteochondral tissues of paired grade 1–2 and grade 3–4 cartilage from 8 randomly selected patients. CD105-positive cells were observed in the superficial cartilage and reticular pattern as well as in the bone marrow cavities of the subchondral bones in both grade 1–2 and grade 3–4 cartilage (Fig. 4c–f, Supplementary Fig. 3). Interestingly, some cartilage matrixes are positive to CD105 staining, which may be explained by the presence of soluble CD105 [36]. Furthermore, more CD105 positive cells were observed in the grade 3–4 superficial cartilage (Fig. 4e) and the bone lining locations near the osteochondral junction area (Fig. 4f), which may suggest that CD105^+^ cells migrate toward and accumulated in damaged cartilages. Moreover, we found a small number of CD271^+^ cells resided in the superficial cartilage of grade 1–2 OA cartilage (Fig. 4g), while more CD271^+^ cells were observed in the superficial cartilage of grade 3–4 OA cartilage (Fig. 4i). Notably, CD271^+^ cells distributed near the osteochondral junction regions and reticular pattern of subchondral bone marrow cavities in both grade 1–2 and grade 3–4 OA subchondral bones (Fig. 4h, j).

### The gene expression profile of mCPCs from grade 1–2 and grade 3–4 OA cartilage

**Fig. 5:**
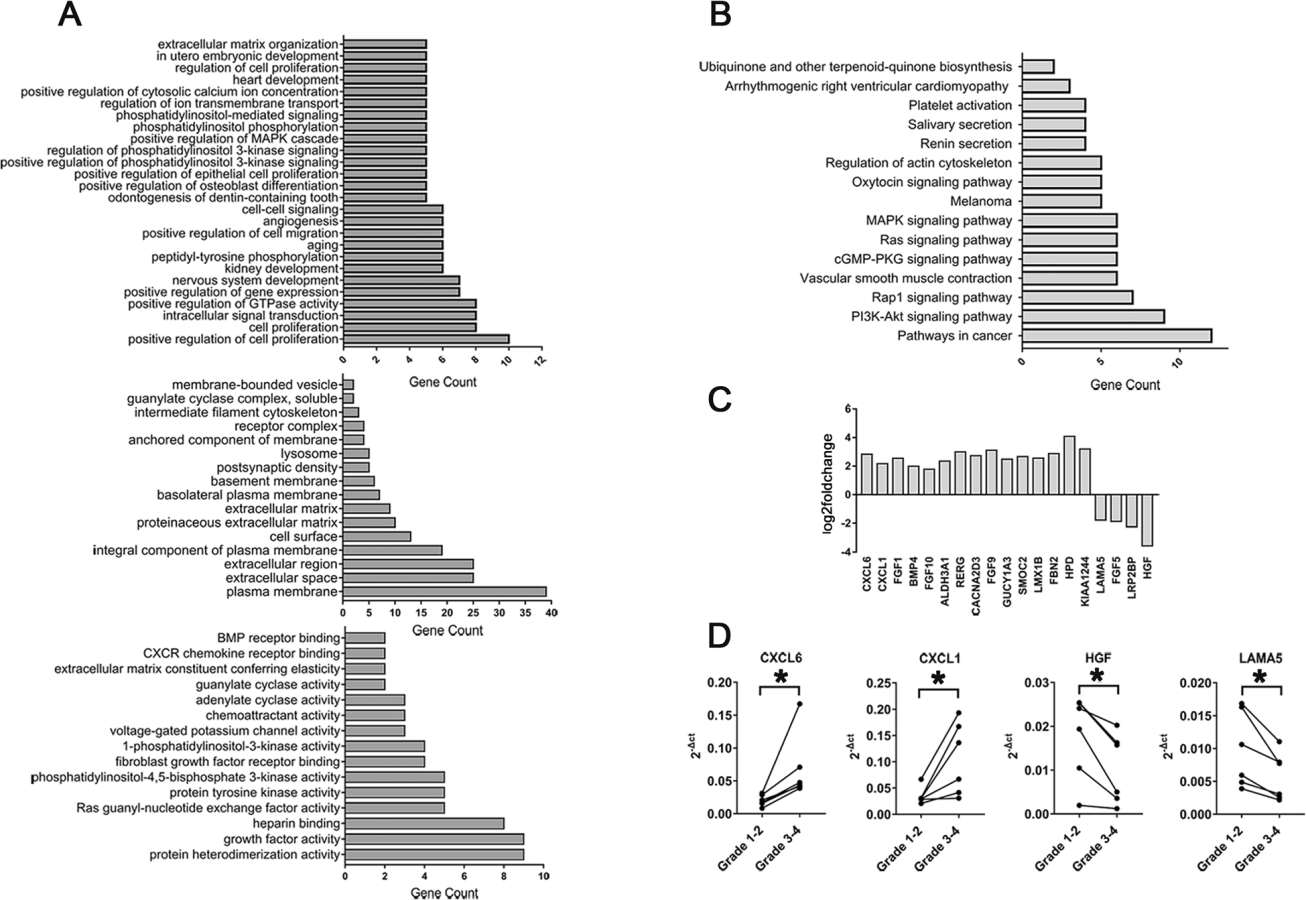
mRNA sequencing analysis of mCPCs from paired grade 1–2 and grade 3–4 OA cartilage. **a** Cluster of significantly changed genes according to various biological processes, cellular components, and molecular function. **b** The differentially expressed genes were showed in the Kyoto Encyclopedia of Genes and Genomes (KEGG) pathways. **c** Histogram showing levels of mean log_2_ fold change in 19 selected relative genes associated with OA pathogenesis in mCPCs from paired grade 3–4 versus grade 1–2 cartilage (*n* = 6 donors). **d** Validation of CXCL6, CXCL1, HGF, and LAMA5 mRNA expression by real-time quantitative polymerase chain reaction; the 2^−△Ct^ value was normalized to β-actin (*n* = 6 donors, with each repeated in triplicates). *p < 0.05, by paired *t* test

The gene expression profile of mCPCs from grade 1–2 and grade 3–4 OA cartilage was analyzed in 6 donors. After normalization, mCPCs from grade 1–2 cartilage were set as log_2_ fold change ≥ 1.0 and *p* < 0.05 to determine the differentially expressed mRNAs. Compared to that of mCPCs from grade 1–2 cartilage, the gene expression of mCPCs in grade 3–4 cartilage indicated that the mRNA expression of at least 134 genes remarkably changed (105 genes upregulated and 29 genes downregulated), including the genes involved in various biological processes, cellular component, molecular function, the expression of some genes related to the cell proliferation and intracellular signal transduction, plasma membrane and extracellular space, protein heterodimerization activity, and growth factor activity (Fig. 5a, b). Nineteen dysregulated genes that are known to be involved in human OA include CXCL6, CXCL1, FGF1, BMP4, FGF10, ALDH3A1, RERG, CACNA2D3, FGF9, GUCY1A3, SMOC2, LMX1B, FBN2, HPD, KIAA1244, LAMA5, FGF5, LRP2BP, and HGF (Fig. 5c). Four dysregulated genes were selected for further validation by RT-qPCR. The results showed that the expressions of genes encoding chemokines proteins (CXCL6 and CXCL1) significantly upregulated, but the expressions of genes encoding growth factor and extracellular matrix (ECM) proteins (HGF and LAMA5) were remarkably decreased (Fig. 5d).

## Discussion

In the present study, we isolated novel subpopulations of CPCs from paired cartilaginous tissues by virtue of their cell migration capacity. We found that mCPCs from Outerbridge grade 1–2 and grade 3–4 cartilage shared similar cell proliferation and self-renewal ability, but the mCPCs (grade 3–4) showed enhanced osteo-adipogenic activities and decreased chondrogenic capacity. Importantly, the mCPCs (grade 3–4) exhibited stronger cell migration in response to OASF. Notably, more CD105^+^/CD271^+^ cells were found resided in grade 3–4 superficial articular cartilages and areas of osteochondral conjunction. Additionally, increased expression of genes encoding chemokines and decreased expression of genes encoding growth factor and extracellular matrix were observed.

The imbalance of extracellular matrix degradation and synthesis in the progress of OA caused by the combination of mechanical and biochemical factors were considered as fundamental factors contributing the destruction of tissue homeostasis. In recent years, increasing attentions have been focused on the fact that the pathological changes of tissue-specific stem cells in articular cartilages which may be closely involved in the development of osteoarthritic diseases. However, inconclusive results were observed in previous studies. An earlier study reported declined potential of CPCs from OA patients [5]. However, another study described that adult CPCs, particularly those from moderately affected regions of the osteoarthritic joints, exhibit superior chondrogenic potential [37]. In addition, the independent studies pursued by Xia et al. and Mantripragada et al. demonstrated that CPCs from the degraded cartilages of the medial condyle and relatively normal cartilages of the lateral side showed similar chondrogenic potential [14, 18, 19].

The inconclusive data might result from the heterogenous CPC isolating protocols and functional investigations in independent labs. First, almost all of the CPCs above were obtained from the released cells post long-time collagenase digestion with either cell colony formation cell expansion [6] or flow cytometry cell sorting [19, 38] except Koelling et al. obtained cells that migrated from human cartilage [5]. Notably, our previous study demonstrated that CPCs migrated from human non-osteoarthritic cartilages represent more regenerative cell subpopulation in cartilages than that of released cells [12]. Second, previous studies showed that the digestion-induced released cells may partially lose their biological functions [20, 21]. In addition, maintaining the bone marrow niche ex vivo in primary culture showed benefits to maintain stem cell properties [23, 39]. In the present study, the cartilage chips were cultivated and retained during cell passaging until passage 3 so as to mimic the CPC niche and allow more CPC outgrowth. Third, osteoarthritic tibial plateau cartilage is usually badly damaged in KOA due to its unique mechanical status. To the best of our knowledge, the differentiation fates of tissue-specific stem/progenitor cells were greatly influenced by mechanical factors. Discher et al. described that a local biochemical and mechanical niche with complex and dynamic regulation control stem cell sense [40]. Yang et al. reported that stem cells remember past physical signals, and mechanical memory and dosing influence stem cell fate [41].

In addition to cell multi-potency, the migratory ability of stem/progenitor cells is essential for cartilage regeneration. It has been reported that both trauma and degenerative lesions activate endogenous CPCs migration by releasing trauma-associated and OA inflammatory factors so as to chemotactically induced CPC migration to injured sites [5, 7, 28, 42]. In the current study, we found that both mCPCs from grade 1–2 and grade 3–4 cartilage showed apparent migration capacity in response to OASF. Interestingly, mCPCs (grade 3–4) exhibited stronger cell migration. The data of CD105^+^ cells in vivo distribution in cartilage may be helpful to understand the phenomenon. The CD105^+^ cell number in the grade 3–4 OA cartilage was remarkably higher than that of the grade 1–2 OA cartilage. In addition, these cells mainly accumulated in superficial cartilages and areas of osteochondral junction. Moreover, CPCs have also been reported to be chemotactic migratory with nerve growth factor (NGF) treatment and result in extracellular matrix catabolism indicated by increased sulfated glycosaminoglycan release and matrix metalloprotease (MMP) expression [43]. Consistently, the results of flow cytometry and histopathological analysis showed higher expression of CD271 in grade 3–4 cartilage-derived mCPCs and in osteo-chondral tissues of grade 3–4 OA specimen, which may contribute to late-stage OA articular cartilage degeneration. However, we are aware that CPCs and other cells including MSCs may share the cell markers in the subchondral bones, and further investigations are needed to find the unique cell markers for CPCs in the future.

To further explore the regulatory genes of osteoarthritic CPCs, we performed an analysis of the gene expression profile of mCPCs from grade 1–2 and grade 3–4 cartilage. Notably, mCPCs (grades 3–4) overall exhibited higher levels of chemokines (CXCL-1, CXCL-6) and lower growth factor (HGF) and ECM protein (LAMA5) than mCPCs (grades 1–2). Previous study have suggested innate associations between OA severity and synovial fluid CXCL1 concentration [44] while the upregulation of CXCL-1 and CXCL-6 is also responsible for stronger migration of mCPCs (grades 3–4). Downregulation of HGF may be responsible for decreased chondrogenic performance of mCPCs (grades 3–4) in cartilage because the previous study have demonstrated that HGF-rich exosome plays a pivotal role in promoting cartilage repair [45]. Also, the downregulated expression of gene LAMA5 has been suggested to hamper the maintenance and function of the ECM which are critical components in stem cell niche. Another study also proved that the heterozygous LAMA5 mutation is closely associated with OA via regulating ECM proteins (COL1A1, MMP1, and MMP3) [46]. Nevertheless, the results of our current mRNA sequencing only showed changes of some genes in CPCs, and further researches are needed to explore underlying molecular mechanisms.

**Fig. 6:**
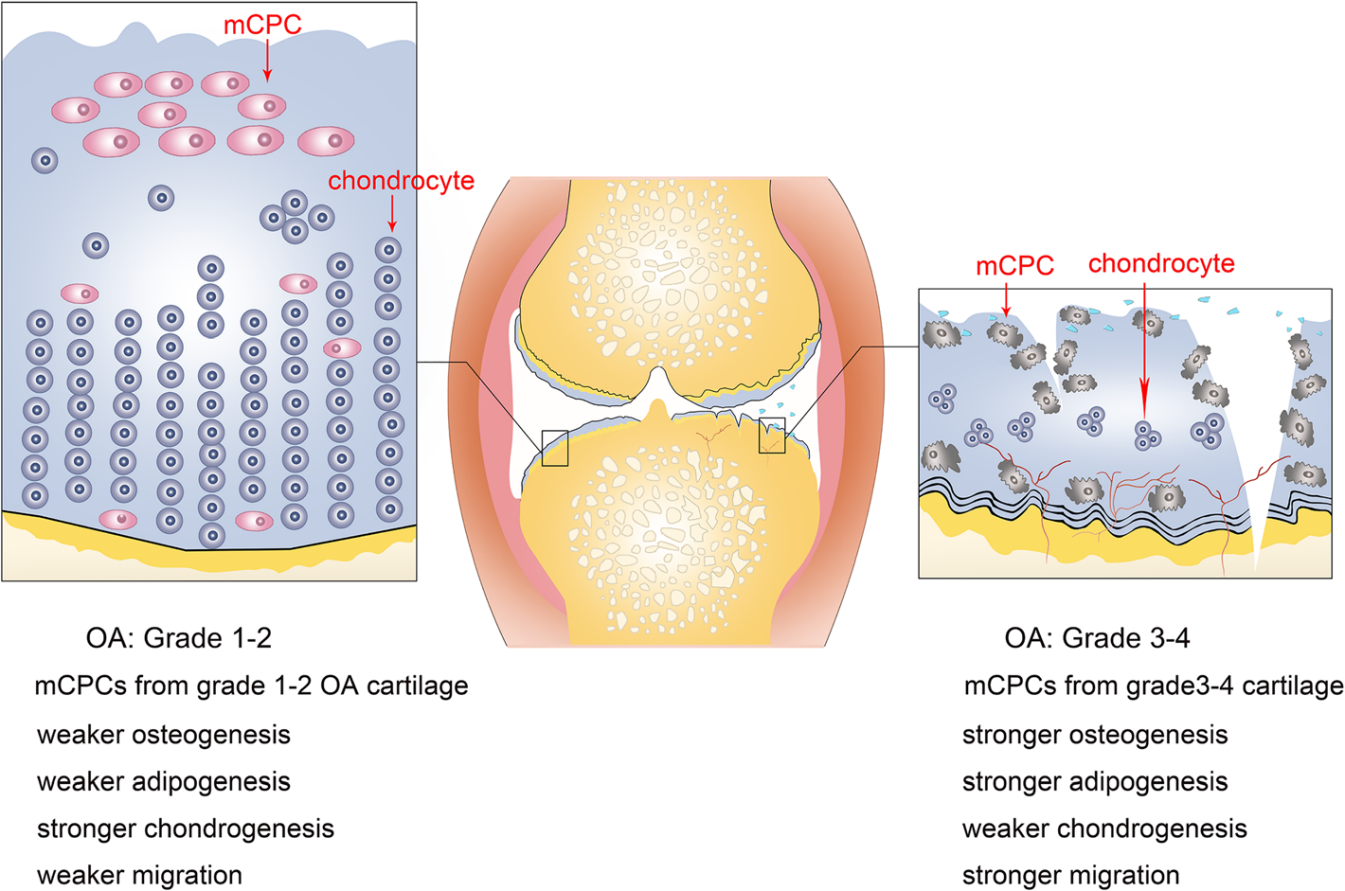
Schematic suggestion of the pathological changes of mCPCs in the progression of OA. In the progressing of OA, more mCPCs migrated into the articular cartilages for tissue repairing. However, the chondrogenerative capacities of mCPCs in grade 3–4 OA cartilage are remarkably impaired. Compared to their counterpart in grade 1–2 OA cartilage, these cells exhibited stronger osteogenesis, stronger adipogenesis, and weaker chondrogenesis

Thus, we speculated pathological changes of mCPCs in the progression of OA (Fig. 6). Although more mCPCs migrated to the degenerative cartilage of lesion sites in the progress of later staged OA, the chondrogenic capacity of these cells are impaired, which changed the self-repairing capacity of articular tissues. Notably, our findings suggested that mCPCs may be optional cell targets for OA treatment. Blocking impaired mCPC migration may delay the articular degeneration. Additionally, rescuing the multi-potency of mCPCs may be helpful to promote tissue repair in later-stage OA.

We acknowledge that there were some limitations in our study. First, the CPCs in grade 1–2 degenerative cartilages are not equated with the fully healthy CPCs. Second, we cannot exclude the possibility that CPC properties were influenced by anatomical (medial-to-lateral or superficial-to-deep) and/or mechanical differences in osteoarthritic cartilage [27]. Third, the cell surface markers of our ex vivo cultured mCPCs is different from that of in vitro culture-expanded [47].

## Conclusions

We have isolated migratory progenitor cell populations from both grade 1–2 and 3–4 human OA cartilages. Although mCPCs in the grade 3–4 OA cartilage present stronger migratory potential, the chondrogenic capacities of these cells are impaired. Our findings may be helpful in understanding the role of mCPCs in the pathogenesis of OA progression.

## Supplementary information

**Additional file 1:Supplementary Figure 1.** Cell proliferation and self-renewal of mCPCs from paired grade 1–2 and grade 3–4 OA cartilage.

**Additional file 2:Supplementary Figure 2.**Migration potential of mCPCs derived from paired grade 1–2 and grade 3–4 cartilage.

**Additional file 3: Supplementary Figure 3.** Negative control for in situ CD105 immunohistochemical distribution of grade 1–2 OA cartilage.

**Additional file 4:Supplementary Table 1.** Demographic, clinical, and imaging characteristics of the donors. **Supplementary Table 2.** Primer sequences for RT-qPCR.

## Data Availability

The datasets generated and analyzed during the current study are available in Figs. 1, 2, 3, 4, 5, and 6; Supplementary Figs. 1, 2, and 3; and Supplementary Tables 1–2. The more detailed datasets are also available from the corresponding author on reasonable request.

## Abbreviations

ALP: Alkaline phosphatase
APC: Allophycocyanin
CCK-8: Cell Counting Kit 8
CEBP/α: CCAAT/enhancer-binding protein alpha
CFU-F: Colony-forming unit fibroblast formation
Col-II: Collagen type II
CPCs: Chondrogenic progenitor cells
DAPI: 4′,6-Diamidino-2-phenylindole
ECM: Extracellular matrix
EDTA: Ethylenediaminetetraacetic acid
FACS: Fluorescence-activated cell sorting
FBS: Fetal bovine serum
FITC: Fluorescein isothiocyanate
KEGG: Kyoto Encyclopedia of Genes and Genomes
KOA: Knee osteoarthritis
mCPCs: Migratory CPCs
MMP: Metalloprotease
MSC: Mesenchymal stem cell
NGF: Nerve growth factor
Sox-9: SRY-type high-mobility group box-9
TGF-β3: Transforming growth factor
OCN: Osteocalcin
OA: Osteoarthritis
PBS: Phosphate-buffered saline
PE: Phycoerythrin
PPARγ: Peroxisome proliferator-activated receptor gamma
RT-qPCR: Real-time quantitative polymerase chain reaction
RUNX2: Runt-related transcription factor 2
SD: Standard deviation
α-MEM: Alpha-minimal essential medium
