# Supplementary figures and images for: Biological potential alterations of migratory chondrogenic progenitor cells during knee osteoarthritic progression
Source: Arthritis Res Ther. 2020 Mar 27;22:62. doi: 10.1186/s13075-020-2144-z (PMC7099802; doi:10.1186/s13075-020-2144-z)

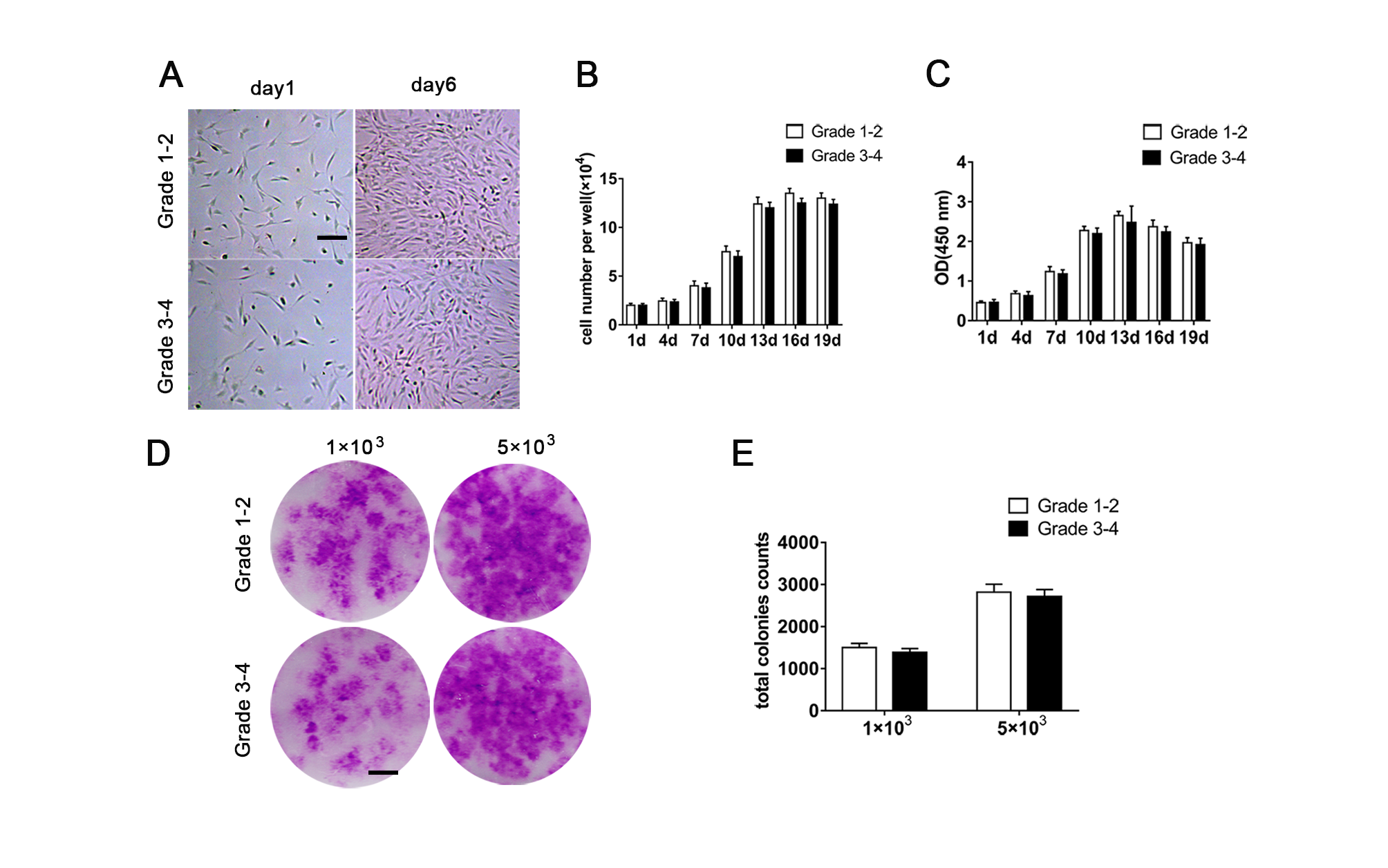

Supplement: Supplementary file 1 — Additional file 1:Supplementary Figure 1. Cell proliferation and self-renewal of mCPCs from paired grade 1–2 and grade 3–4 OA cartilage. [file 13075_2020_2144_MOESM1_ESM.tif]

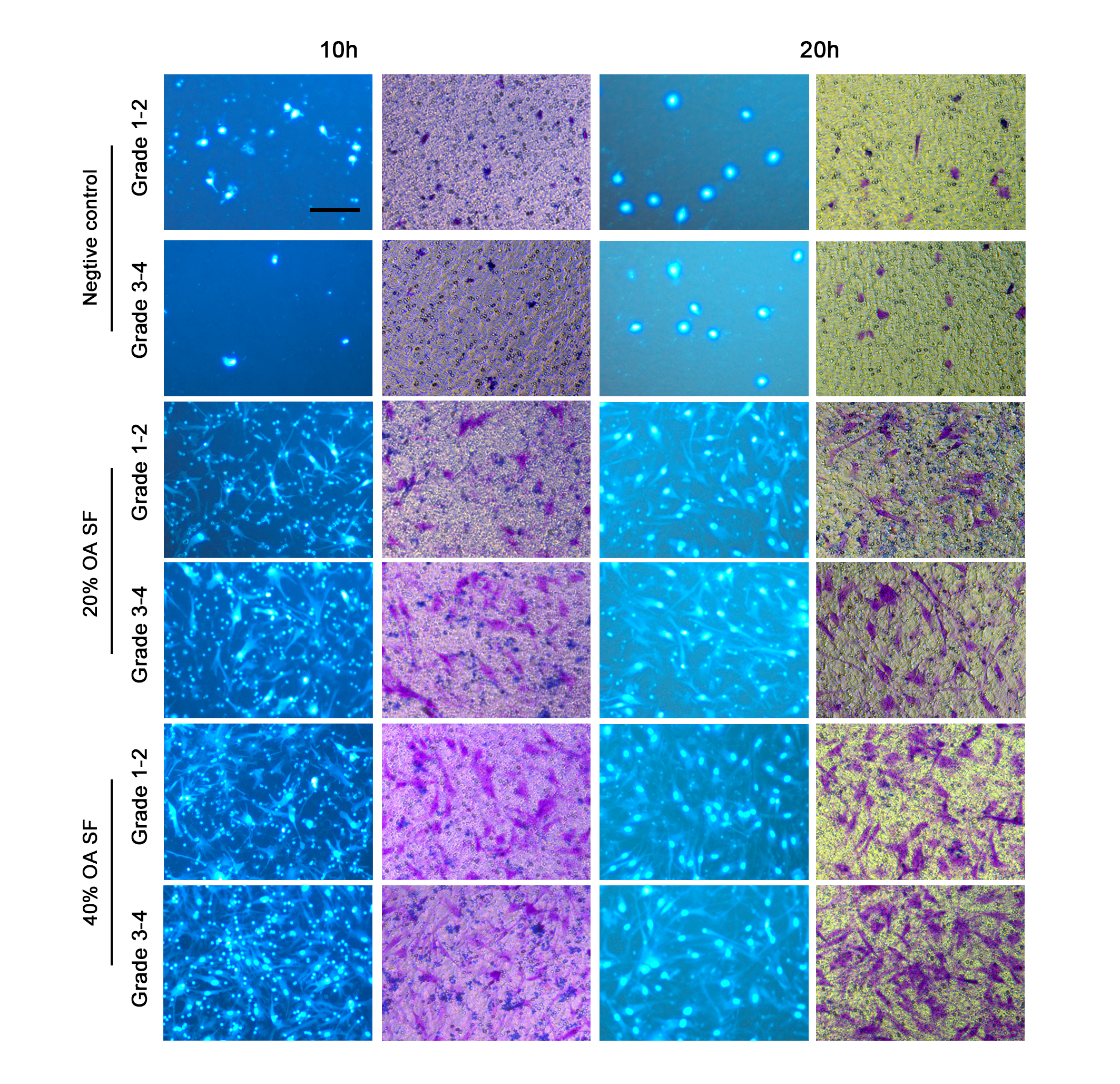

Supplement: Supplementary file 2 — Additional file 2:Supplementary Figure 2.Migration potential of mCPCs derived from paired grade 1–2 and grade 3–4 cartilage. [file 13075_2020_2144_MOESM2_ESM.tif]

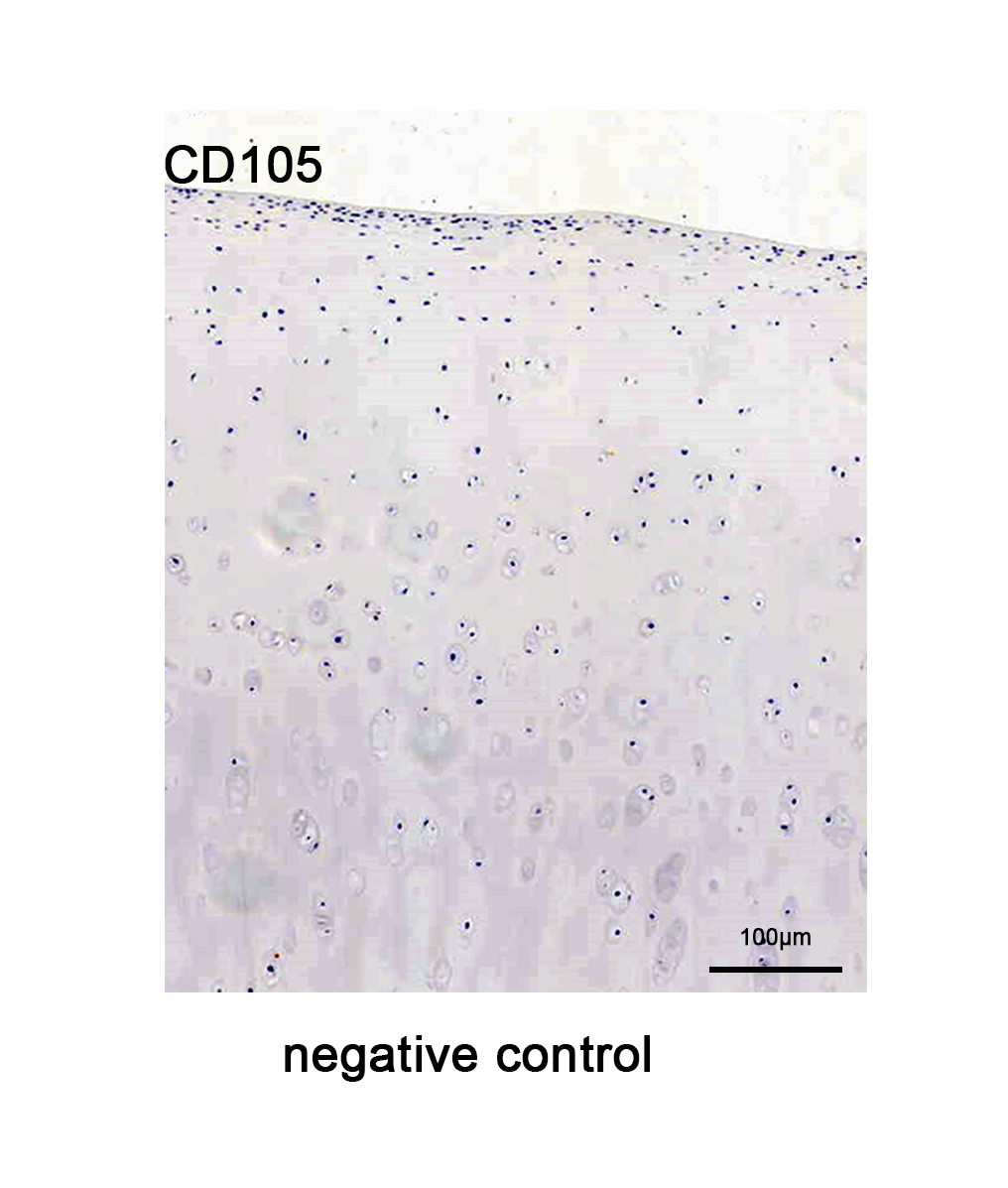

Supplement: Supplementary file 3 — Additional file 3: Supplementary Figure 3. Negative control for in situ CD105 immunohistochemical distribution of grade 1–2 OA cartilage. [file 13075_2020_2144_MOESM3_ESM.tif]
