# Supplementary material for: Biological potential alterations of migratory chondrogenic progenitor cells during knee osteoarthritic progression
Source: Arthritis Res Ther. 2020 Mar 27;22:62. doi: 10.1186/s13075-020-2144-z (PMC7099802; doi:10.1186/s13075-020-2144-z)
Supplement: Supplementary file 4 — Additional file 4:Supplementary Table 1. Demographic, clinical, and imaging characteristics of the donors. Supplementary Table 2. Primer sequences for RT-qPCR. [file 13075_2020_2144_MOESM4_ESM.docx]

**Supplementary Table 1. Demographic, clinical, and imaging characteristics of the donors.**

| ID | Age  (years) | Sex | BMI  (kg/m^2^) | Disease  duration  (years) | cartilage scores for  MFTJ (using WORMS) | cartilage scores for  LFTJ(using WORMS) |
| --- | --- | --- | --- | --- | --- | --- |
| 1 | 72 | M | 24.4 | 8 | 21 | 4 |
| 2 | 63 | F | 25.6 | 9 | 20 | 4 |
| 3 | 73 | F | 26.7 | 11 | 20.5 | 3 |
| 4 | 69 | M | 24.8 | 9 | 21.5 | 5 |
| 5 | 55 | F | 25.5 | 3 | 21 | 8 |
| 6 | 61 | F | 25.1 | 4 | 18.5 | 9 |
| 7 | 57 | F | 30.8 | 8 | 24 | 7 |
| 8 | 53 | F | 23.6 | 5 | 19 | 6 |
| 9 | 58 | M | 24.4 | 4 | 21 | 5 |
| 10 | 56 | F | 27.3 | 7 | 20 | 3 |
| 11 | 66 | F | 28.4 | 7 | 23 | 10 |
| 12 | 56 | M | 25.3 | 5 | 16.5 | 4 |
| 13 | 63 | F | 27.8 | 8 | 20 | 6 |
| 14 | 70 | F | 23.7 | 9 | 18 | 7 |
| 15 | 53 | F | 22.7 | 4 | 12 | 5 |
| 16 | 68 | F | 26.5 | 8 | 16 | 5 |
| 17 | 70 | M | 27.8 | 11 | 18 | 8 |
| 18 | 68 | F | 27.6 | 15 | 25 | 10 |
| 19 | 65 | M | 30.3 | 10 | 19 | 9 |
| 20 | 68 | F | 23.5 | 6 | 15.5 | 7 |
| 21 | 64 | F | 23.9 | 7 | 21 | 5 |
| 22 | 59 | F | 28.7 | 8 | 22 | 6 |
| 23 | 68 | F | 25.3 | 8 | 20 | 4 |
| 24 | 65 | M | 25.4 | 5 | 16.5 | 5 |
| 25 | 68 | F | 28.3 | 8 | 22 | 9 |
| 26 | 69 | M | 26.3 | 5 | 14 | 8 |
| 27 | 64 | M | 23.8 | 5 | 19 | 5 |
| 28 | 59 | F | 24.2 | 6 | 18 | 6 |

BMI: body mass index; WORMS: whole-organ MRI scoring; MFTJ: medial femorotibial joint; LFTJ: lateral femorotibial joint.

**Supplementary Table 2. Primer sequences for RT-qPCR**

| genes | primer sequences |
| --- | --- |
| Runx-2 | forward, 5’- CTGTGGTTACTGTCATGGCG-3′  reverse, 5’- CCCTCCCTTTTCCCACTCAT-3′ |
| OCN | forward, 5′- AGGTGCGAAGCCCAGCGGTGCA-3′  reverse, 5′- CCTGGAGAGGAGCAGAACTGGG-3′ |
| CEBP/α | forward, 5′- GGAGGGTCTCTAGTTCCACG-3′  reverse, 5′- CCCACAGCCAGATCTCTAGG-3′ |
| PPARγ | forward, 5′- TTGCAGTGGGGATGTCTCAT-3′  reverse, 5′- TTTCCTGTCAAGATCGCCCT-3′ |
| Sox-9 | forward, 5′- ATGAAGATGACCGACGAGCA -3′  reverse, 5′- AACTTGTCCTCCTCGCTCTC-3′ |
| Col-II | forward, 5′- AGCCTGGTGATGATGGTGAA-3′  reverse, 5′-ACTCTCACCCTTCACACCAG-3′ |
| CXCL1 | forward, 5′-ACTCTACCTGCACACTGTCC-3′  reverse, 5′-TCCCCTGCCTTCACAATGAT-3′ |
| CXCL6 | forward, 5′-CGAACCCTCTCTTGACCACT-3′  reverse, 5′-TTGGGGTTTACTCTCAGCGT-3′ |
| HGF | forward, 5′-TGGTAAAGGACGCAGCTACA-3′  reverse, 5′-GCGTACCTCTGGATTGCTTG-3′ |
| LAMA5 | forward, 5′-CTGTGCAAACCCAACTTCCA-3′  reverse, 5′-CTGGCAGAGAGGGAAGTGAA-3′ |
| β-actin | forward, 5′AGCACAATGAAGATCAAGATCAT-3′  reverse, 5′-ACTCGTCATACTCCTGCTTGC-3′ |
| RPL13a | forward, 5′-GCCATCGTGGCTAAACAGGTA-3′  reverse, 5′-GTTGGTGTTCATCCGCTTGC-3′ |

RT-qPCR: real-time quantitative polymerase chain reaction; Runx-2: runt-related transcription factor 2; OCN: osteocalcin; CEBP/α: CCAAT/enhancer binding protein alpha; PPARγ: peroxisome proliferator-activated receptor gamma; Sox-9: sex determining region Y-box 9; Col-II: type II collagen, RPL13a: ribosomal protein L13a.
